# High CD8+ T Cell Activation Marks a Less Differentiated HIV-1 Specific CD8+ T Cell Response that Is Not Altered by Suppression of Viral Replication

**DOI:** 10.1371/journal.pone.0004408

**Published:** 2009-02-09

**Authors:** Jason D. Barbour, Lishomwa C. Ndhlovu, Qi Xuan Tan, Terence Ho, Lorrie Epling, Barry M. Bredt, Jay A. Levy, Frederick M. Hecht, Elizabeth Sinclair

**Affiliations:** 1 HIV/AIDS Division, San Francisco General Hospital, Department of Medicine, University of California San Francisco, San Francisco, California, United States of America; 2 Division of Experimental Medicine, San Francisco General Hospital, Department of Medicine, University of California San Francisco, San Francisco, California, United States of America; 3 UCSF Core Immunology Laboratory, San Francisco General Hospital, Department of Medicine, University of California San Francisco, San Francisco, California, United States of America; New York University School of Medicine, United States of America

## Abstract

**Background:**

The relationship of elevated T cell activation to altered T cell differentiation profiles, each defining features of HIV-1 infection, has not been extensively explored. We hypothesized that anti-retroviral suppression of T cell activation levels would lead to alterations in the T cell differentiation of total and HIV-1 specific CD8+ T cell responses among recently HIV-1 infected adults.

**Methodology/Principal Findings:**

We performed a longitudinal study simultaneously measuring T cell activation and maturation markers on both total and antigen-specific T cells in recently infected adults: prior to treatment; after the initiation of HAART; and after treatment was halted. Prior to treatment, HIV-1 Gag–specific CD8+ T cells were predominantly of a highly activated, intermediate memory (CD27+CD28−) phenotype, while CMV pp65-specific CD8+ T cells showed a late memory (CD27−CD28−), low activation phenotype. Participants with the highest fraction of late memory (CD27−CD28−) HIV-1-specific CD8+ T cells had higher CD4+ T cell counts (rho = +0.74, p = 0.004). In turn, those with the highest fraction of intermediate memory (CD27+ CD28−) HIV-1 specific CD8+ T cells had high total CD8+ T cell activation (rho = +0.68, p = 0.01), indicating poorer long-term clinical outcomes. The HIV-1 specific T cell differentiation profile was not readily altered by suppression of T cell activation following HAART treatment.

**Conclusions/Significance:**

A more differentiated, less activated HIV-1 specific CD8+ T cell response may be clinically protective. Anti-retroviral treatment initiated two to four months after infection lowered T cell activation but had no effect on the differentiation profile of the HIV-1-specific response. Intervention during the first month of acute infection may be required to shift the differentiation phenotype of HIV-1 specific responses to a more clinically favorable profile.

## Introduction

Elevated CD8+ T cell activation is established early in HIV-1 infection [1], is a hallmark of HIV-1 disease [2] and predicts subsequent poor clinical outcome in a manner independent of viral load [1]. Furthermore, elevated CD8+ T cell activation, and not viral load, distinguish pathogenic from non-pathogenic lentiviral infections [3]–[6]. These observations have lead to a general consensus that CD8+ T cell activation plays an important role in the pathogenesis of immunodeficiency. Yet the mechanism whereby elevated CD8+ T cell activation leads to immunodeficiency during untreated HIV-1 disease remains unclear. The T cell response to HIV-1 is marked by a second distinguishing feature. Several groups have observed that HIV-1 specific CD8+ T cells fail to differentiate to a fully mature effector cell [7], [8]. We hypothesized that a block in T cell maturation may be related to elevated activation on HIV-1 specific CD8+ T cells, and be relieved by suppression of T cell activation levels by anti-retroviral treatment (ART).

On encountering antigen CD8+ T cells differentiate from the least differentiated (naïve or early memory) stage to the most mature (memory/effector) stage. In this process, cell surface receptors are progressively down-regulated (CD45RA CCR7, CD28, CD27, CD127) as CD8+ T cells differentiate and up-regulated or re-expressed (CD57, CD45RA). While there is broad general agreement on how to define a naïve T cell, there is not yet a unified model that describes the process of human T cell differentiation (reviewed by Appay et al 2008 [9]) but memory cells can be divided into Early (EM), Intermediate (IM) and Late (LM) stages using CD27 and CD28. T cell differentiation profiles have been found to differ across antigen specificities, and may relate to the effectiveness of long-term control [10], [11]. The CMV-specific CD8+ T cell response displays a predominantly late memory phenotype. As CMV infection is typically well controlled in adults, this suggests a more differentiated phenotype may be beneficial [12]. The reverse is true for HIV-1-specific CD8+ T cells, which are enriched for a more intermediate memory phenotype. Likewise, in chronic, virulent HCV infection there is an expansion of the intermediate memory and a lack of late memory, HCV specific CD8+ cells [13]. Among EBV/HIV-1 co-infected persons, the absence of differentiated EBV specific CD8+ T cells has been associated with EBV-associated non-Hodgkins lymphoma [8].

As yet there has been limited work measuring the simultaneous expression of activation and differentiation markers on HIV-specific T cells [14]–[16]. Here, we used polychromatic flow cytometry to examine the co-expression of CD38, CD27 and CD28 on total T cells and antigen-specific (HIV, CMV) T cells in a cohort of adults in early HIV-1 infection (within 6 months of acquisition). This approach allowed us to examine the relationship between T cell activation and differentiation, relate these profiles to HIV clinical markers among recently HIV-1 infected adults, and determine if effective anti-retroviral therapy can durably alter these profiles. We observed that HIV-1 Gag-specific CD8+ T cells were predominantly of an IM phenotype and were significantly more activated than LM Gag-specific CD8+ T cells. Individuals with the highest CD8+ T cell activation levels also had the greatest proportion of IM Gag-specific CD8+ T cells while those with the highest proportion of more differentiated LM Gag-specific CD8+ T cells had the highest CD4+ T cell counts. Together these data suggest that the presence of mature Gag-specific CD8+ T cells with low levels of activation may be protective during early infection. Our analysis may serve as a resource to researchers who seek to understand the relationship of key T cell markers to one another, as part of an effort to describe an effective T cell response to HIV-1.

## Results

Demographic, laboratory and clinical markers at all three study time-points (pre-therapy baseline visit 1, on anti-retroviral therapy visit 2, and post-anti-retroviral therapy visit 3) are shown in Table 1.

**Table 1:** Demographic, Clinical and Laboratory Measures By Study Time-point

	Study Entry	On Treatment*		Post-Treatment	
Measurement					
N	13	11		11	
	Median (IQR)		P = **		P = **
CD4+T cell count (cells/µL)	522 (490, 671)	899 (612, 1170)	0.004	715 (579, 1181)	0.04
Viral Load (log_10_ c/mL)	4.95 (4.1, 5.7)	2.31 (2.31, 2.31)	0.004	4.05 (2.87, 4.89)	0.05
CD8+ T cell Activation (% CD38, HLA-DR)	56.9 (37.5, 75.1)	21.1 (15.7, 29.4)	0.0002	37.5 (30.7, 57.1)	0.08
CD38 MFI, HIV-1 Gag IFN-γ+ CD8+ T cells	16825 (5523, 29874)	1614 (1057, 2697)	0.0002	6618 (3053, 7279)	0.03
	16825 (5523, 29874)				
HIV-1 Gag specific IFN-γ+ CD8+ T cells (%)	0.22 (0.14, 1.11)	0.34 (0.26, 0.47)	0.54	0.76 (0.61, 1.88)	0.08
CMV pp65 specific IFN-γ+ CD8+ T cells (%)	1.68 (0.19, 2.57)	1.6 (0.24, 3.19)	0.34	2.65 (1.49, 4.40)	0.002
Age (Years)	32 (30, 39)				
Estimated Length of Infection (Weeks)	10 (7, 12)				
Weeks From Study Entry		28 (18.2, 4.5)			
Weeks Spent on Anti-Retroviral Treatment*				49.2 (19.4, 66.3)	
Weeks Anti-Retroviral Treatment Stopped				13.4 (4.4, 24.1)	
Ethnicity					
White	11 (85%)				
Hispanic	2 (15%)				
Male Gender	13 (100%)				
CMV Positive	12 (92%)				

* Treatment (anti-retroviral therapy) is defined as at least 2 nucleoside reverse transcriptase inhibitors, and either 1 protease inhibitor (PI), 1 non-nucleoside reverse transcriptase inhibitor (nnRTI), or a PI and a nnRTI. All patients were treatment naïve at study entry.

** Difference from Study Entry Values (Sign Rank Test).

### Magnitude of T cell responses to HIV-1 Gag and CMV pp65 over time

The magnitude of IFN-γ and IFN-γ/IL-2 CD8+ T cell responses to HIV-1 Gag were lower than the response to CMV pp65 at visit 1 (prior to therapy), and subsequent study time points during and after anti-retroviral therapy (Fig. 2). The magnitude of Gag IFN-γ CD8+ T cell responses declined during virologically suppressive anti-retroviral therapy, while the magnitude of responses for CMV pp65 did not change.

**Figure 1:**
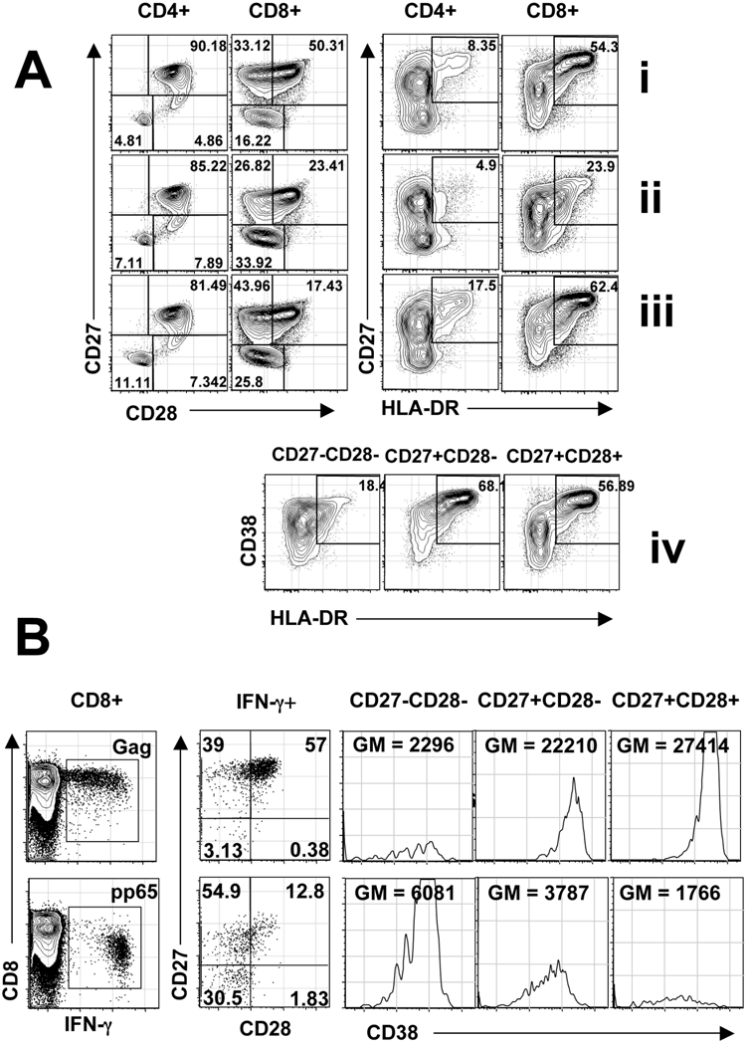
Expression of Maturation and Activation markers on Total CD4+ and CD8+ T cells. (A): before initiation of ART (i); after treatment (ii); and post-ART (iii). Activation is also shown on CD27 and CD28 subsets from CD8+ T cells post-ART (iv). Expression of Maturation and Activation markers on HIV Gag- and CMV pp65-specific T cells (B). CD27 and CD28 expression and CD38 mean fluorescent intensity (MFI) is shown on IFN-γ+ CD8+T cells. (GM = Geometric Mean).

**Figure 2:**
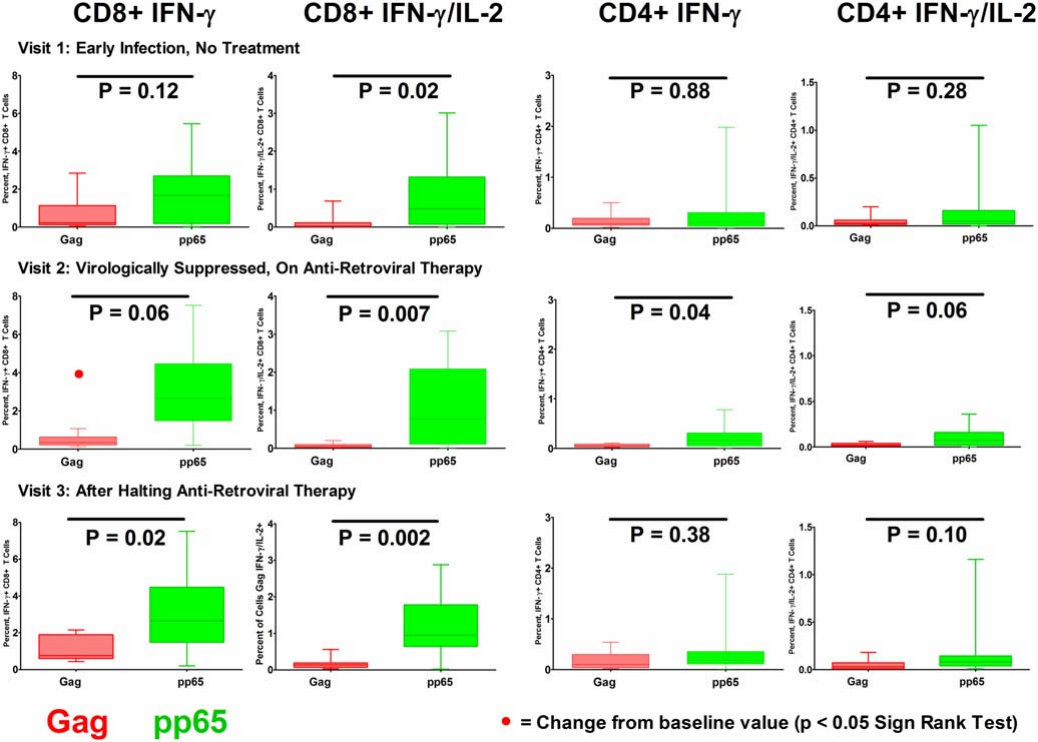
Magnitude of CD4+ and CD8+ T Cell Responses to HIV-1 Gag and CMV pp65. at each of the three study visits. Proportion of IFN-γ+ and IFN-γ+IL-2+ CD8+ (columns 1 and 2) and CD4+ (columns 3 and 4) T cells, responding to stimulation with Gag and pp65 peptide pools. Results for visit 1, prior to antiretroviral therapy visit 2, during a virologically suppressive anti-retroviral regimen, and visit 3, after patients had halted an anti-retroviral regimen and viremia had rebounded are shown in upper, middle and lower rows respectively. Black bars indicate differences between HIV-1 Gag and CMV pp65 responses by response category (Wilcoxon 2 Sample Test). Red dot (1^st^ column, 2^nd^ row) notes a significant change from baseline (visit 1) values (Sign Rank Test). Only the CD8+ T cell IFN-γ response to Gag was observed to significantly change (in this case decline) from visit 1 to either visit 2 or visit 3.

### Differentiation

#### Differentiation of Total T cell subsets

**Figure 3:**
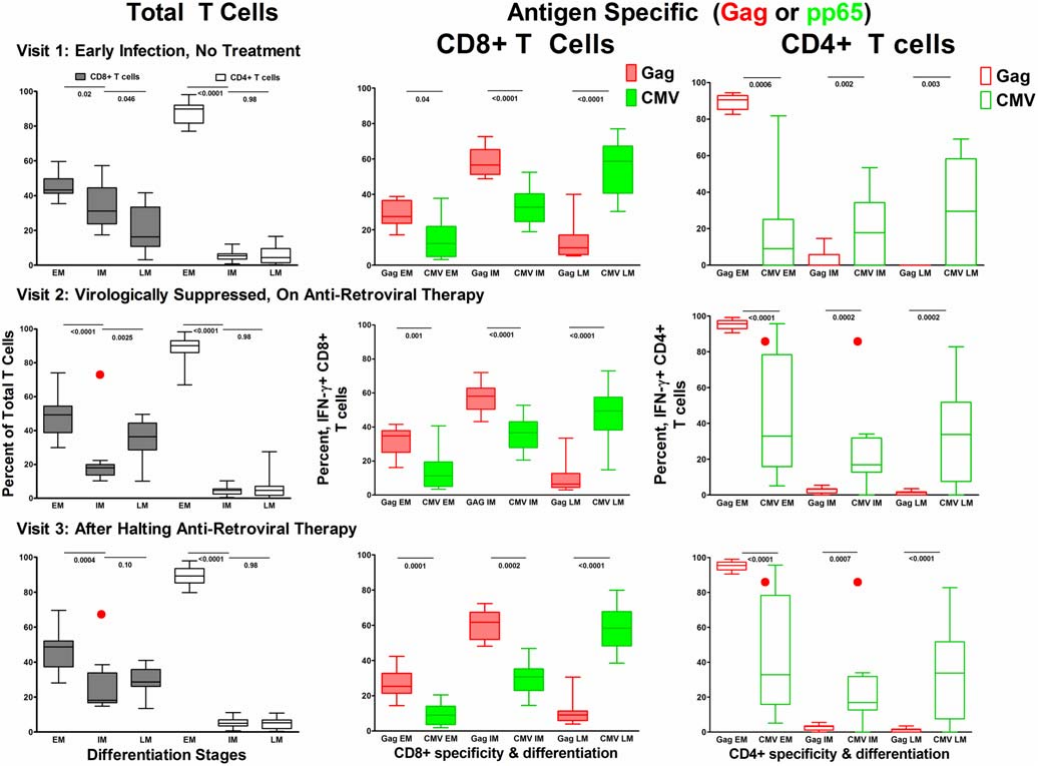
Differentiation profile of total and antigen specific T cells over time. In the first column the proportion of total T cells (CD8+ and CD4+) falling into each T cell maturation category are displayed and defined as early memory (CD27+CD28+) EM, intermediate memory (CD27+CD28−) IM, and late memory (CD27−CD28−) LM. In columns 2 the maturation profiles for CD8+ T cell IFN-γ responses to both HIV-1 Gag (red) and CMV pp65 (green) are shown. And in column 3 the maturation profiles for CD4+ T cell IFN-γ responses to HIV-1 Gag (red) and CMV pp65 (green) are shown. Row 1 displays measurements for visit 1, prior to antiretroviral therapy. Row 2 displays measurements for visit 2, during a virologically suppressive anti-retroviral regimen, and row 3 displays measurements for visit 3, after patients had halted an anti-retroviral regimen and viremia had rebounded. Black bars indicate differences between HIV-1 Gag and CMV pp65 responses by response category (Wilcoxon 2 Sample Test). Red dots note a significant change from baseline (visit 1) values (Sign Rank Test).

At visit 1, prior to anti-retroviral therapy, the naïve and early memory CD8+ T cell subset (EM:CD27+CD28+) was most frequent (median 43.3% Interquartile range (IQR) 41.4, 49.7) followed by the intermediate memory (IM:CD27+CD28−, median 31.20% (IQR 23.8, 44.6)) and late memory (LM:CD27−CD28−) median 16.3% (IQR 10.8, 33.4)) subsets (Fig. 3). Following initiation of anti-retroviral therapy the total CD8+ T cell population shifted out of the intermediate memory CD27+CD28− phenotype, with a greater fraction now falling in the early memory CD27+CD28+ CD8+ T cell population. Once therapy was halted there was a partial but incomplete shift towards an expansion of the intermediate memory pool, but this fraction remained below pre-treatment levels. The total CD4+ T cell differentiation profile did not shift in response to treatment.

#### Differentiation phenotype of HIV-1 Gag- and CMV pp65-specific T cells

We observed that the maturation profiles of antigen-specific T cells differed from the profiles of total T cell populations. HIV-1 Gag-specific IFN-γ expressing CD8+ T cells were predominantly CD27+CD28−, IM (56.6% (IQR 51.3, 65.3)), while the majority of CMV pp65-specific CD8+ T cells had a LM CD27−CD28− phenotype (58.7% (40.7, 67.2)) (Fig. 3). HIV-1 Gag-specific CD4+ T cells were almost entirely in the EM CD27+CD28+ pool, while a greater fraction of pp65-specific CD4+ T cells were in the LM CD27−CD28− pool (Fig. 3). The maturation profile of HIV-1 Gag-specific and CMV pp65-specific CD8+ T cell responses did not change after the initiation of anti-retroviral therapy, or once therapy was halted (Fig. 3). In response to therapy the proportion of CMV pp65-specific CD4+ T cells in the CD27+CD28− fraction declined and a co-responding increase was seen in the CD27+CD28+ fraction, a change which remained once therapy was halted.

### Activation

#### Activation of EM, IM and LM subsets of Total CD8+ and CD4+ T cells

**Figure 4:**
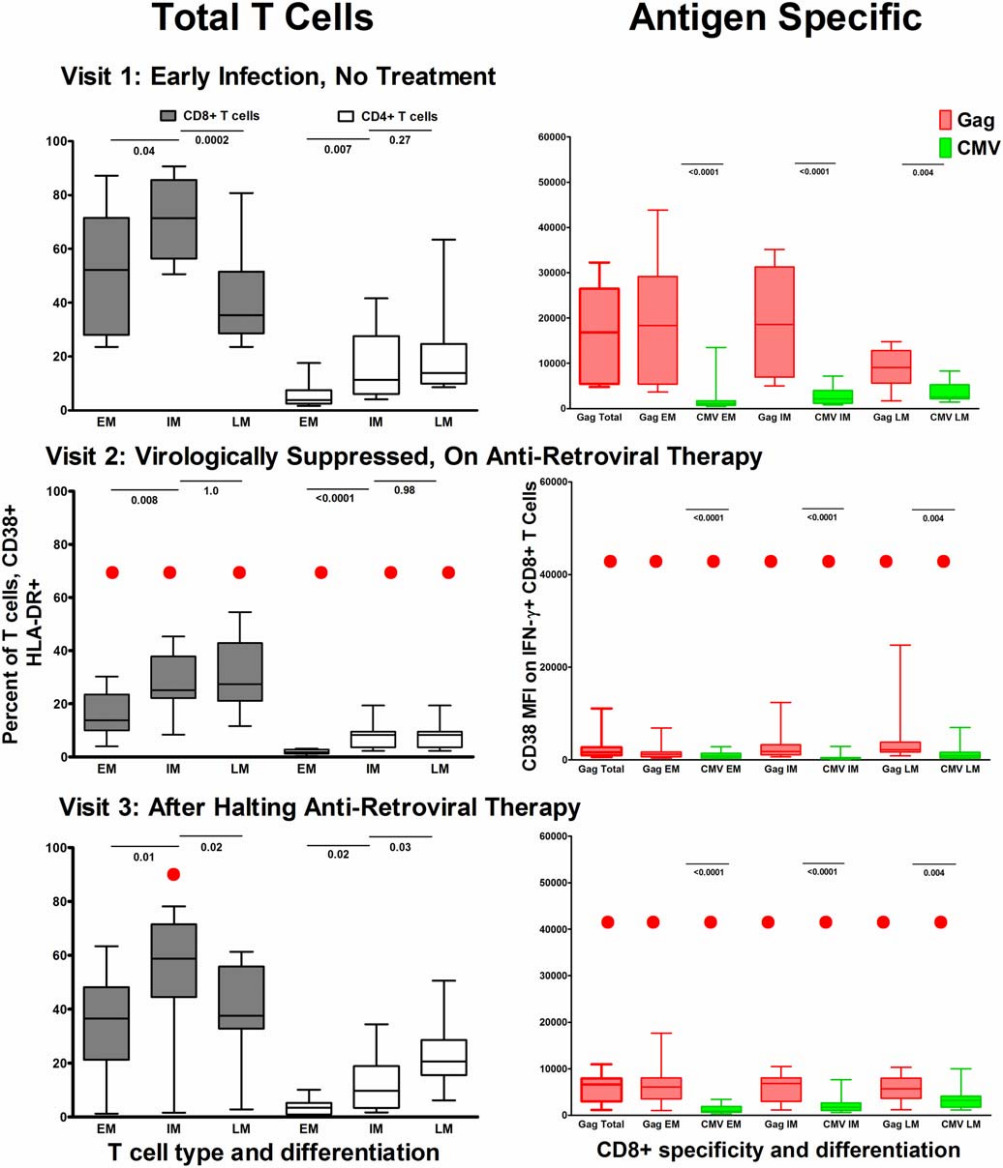
Activation levels on total and antigen specific T cells by differentiation stage. In the first column the proportion of total T cells (CD8+ and CD4+) expressing CD38 and HLA-DR activation markers by T cell maturation category are displayed and defined as early memory (CD27+CD28+) EM, intermediate memory (CD27+CD28−) IM, and late memory (CD27−CD28−) LM. In columns 2 the CD38 MFI level is shown by maturation stage for CD8+ T cell IFN-γ responses to both HIV-1 Gag (red) and CMV pp65 (green). And in column 3 the CD38 MFI is shown for each maturation stage of CD4+ T cell IFN-γ responses to HIV-1 Gag (red) and CMV pp65 (green). Row 1 displays measurements for visit 1, prior to antiretroviral therapy. Row 2 displays measurements for visit 2, during a virologically suppressive anti-retroviral regimen, and row 3 displays measurements for visit 3, after patients had halted an anti-retroviral regimen and viremia had rebounded. Black bars indicate differences between activation levels by response categories (Wilcoxon 2 Sample Test). Red dots notes a significant change from baseline (visit 1) values (Sign Rank Test).

Typical of early HIV-1 infection, the proportion of activated CD8+ T cells was high (median CD38+HLA-DR+ 56.9%, Table 1), and was highest among the IM CD27+CD28− pool (median CD38+HLA-DR+ 71.4% (56.6, 85.6), Figure 4). The proportion of EM CD27+CD28+ (naïve and early memory) CD8+ T cells that were activated was variable between subjects (median CD38_HLA-DR+ 52.2% (28, 71.5) (Fig. 4). The LM CD27+CD28− subset had the lowest proportion of activated cells (median CD38+DR+ 35.3% (IQR 28.7, 51.6)) (Fig. 4). The median proportion of activated total CD4+ T cells was considerably lower than CD8+ T cells (Fig. 4).

#### T cell activation and differentiation state of antigen-specific CD8+ T cell populations

At visit 1, prior to treatment, Gag-specific CD8+ T cells were significantly more activated (higher CD38 MFI) than CMV pp65-specific CD8+ T cells; this was also true for each of the maturation subsets (EM,IM,LM) (Fig. 4). Of the Gag-specific CD8+ T cell subsets the EM and IM populations had a similar, though highly variable level of activation (median EM CD38 MFI, 18340 (IQR 5401, 29205); median IM CD38 MFI, 18566 (IQR 6950, 31203)) while activation was lowest on the most mature LM subset (median CD38 MFI, 9071 (IQR 5613, 12819)) (Fig. 4).

#### Relationship of T cell activation to maturation profile of T cell populations

**Figure 5:**
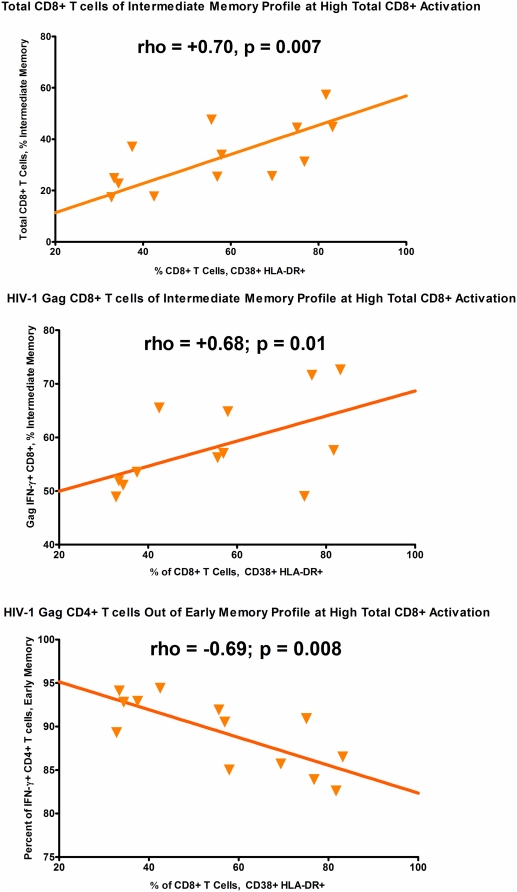
Relationship of Total CD8+ T Cell Activation to T Cell Maturation Profiles. Relationship between total CD8+ T cell activation levels at visit 1 (pre-treatment) on the x-axis and the maturation profile of CD8+ and CD4+ T cell populations, total or antigen specific. In the first panel total CD8+ T cell activation is higher when a greater fraction of total CD8+ T cells fall in the intermediate memory (CD27+CD28−) phenotype. In the second panel total CD8+ T cell activation levels are higher when a greater fraction of HIV-1 Gag specific CD8+ T cells fall in the intermediate memory (CD27+CD28−) phenotype. In the third panel total CD8+ T cell activation levels are lowest when a lower fraction of HIV-1 Specific CD4+ T cells fall into the early memory (CD27+CD28+) phenotype.

At study visit 1, during early untreated HIV-1 infection, we examined the relationship between the CD8+ T cell activation level and maturation profile of total and HIV-1 Gag specific T cell responses (Figure 5). Among untreated persons at this early infection time-point with high CD8+ T cell activation levels, we observed a greater fraction of both total and HIV-1 gag specific CD8+ T cells fall into the intermediate memory (CD27+CD28−) phenotype (figure 5). There was no relationship between the proportion of CD8+ T cells that were activated and either the early memory or late memory fractions of total or Gag-specific CD8+ T cells (data not shown). There was also no relationship between the proportion of CD8+ T cells that were activated and the early, intermediate or late memory fractions of total or Gag-specific CD4+ T cells. For CD4+ T cell responses, at high CD8+ T cell activation levels we did observe that a smaller fraction of HIV-1 Gag specific CD4+ T cells fell into the early memory (CD27+CD28+) pool. There were insufficient cells in the IM and LM CD4+ Gag specific pools to evaluate their relationship with CD8+ T cell activation. We did not observe any relationship between the proportion of activated CD8+ T cells and the size of CMV-specific early, intermediate or late memory fractions.

#### Activation, Maturation and Clinical Disease Markers

**Figure 6:**
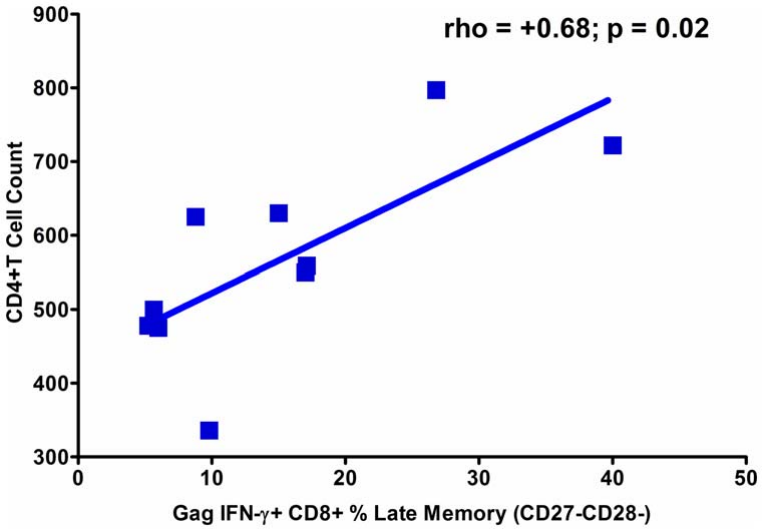
A More Differentiated Gag Specific CD8+ T Cell Response is Protective in Early HIV-1 Infection. Spearman Correlation results shown. Correlation performed on data from pre-treatment, Visit 1. Patients with higher CD4+ T cell counts during early infection and prior to anti-retroviral therapy tended to have higher HIV-1 Gag specific IFN-γ late memory (CD27−CD28−) CD8+ T cells

The size of any differentiation stage of the HIV-1 Gag IFN-γ+ CD8+ T cell response was not associated with viral load (not shown). A higher fraction of HIV-1 Gag specific IFN-γ expressing CD8+ T cells in the LM pool was found to associate with higher CD4+ T cell counts (Fig. 6).

#### Summary of Longitudinal Effects of Anti-Retroviral Therapy on Observed Phenotypes

**Figure 7:**
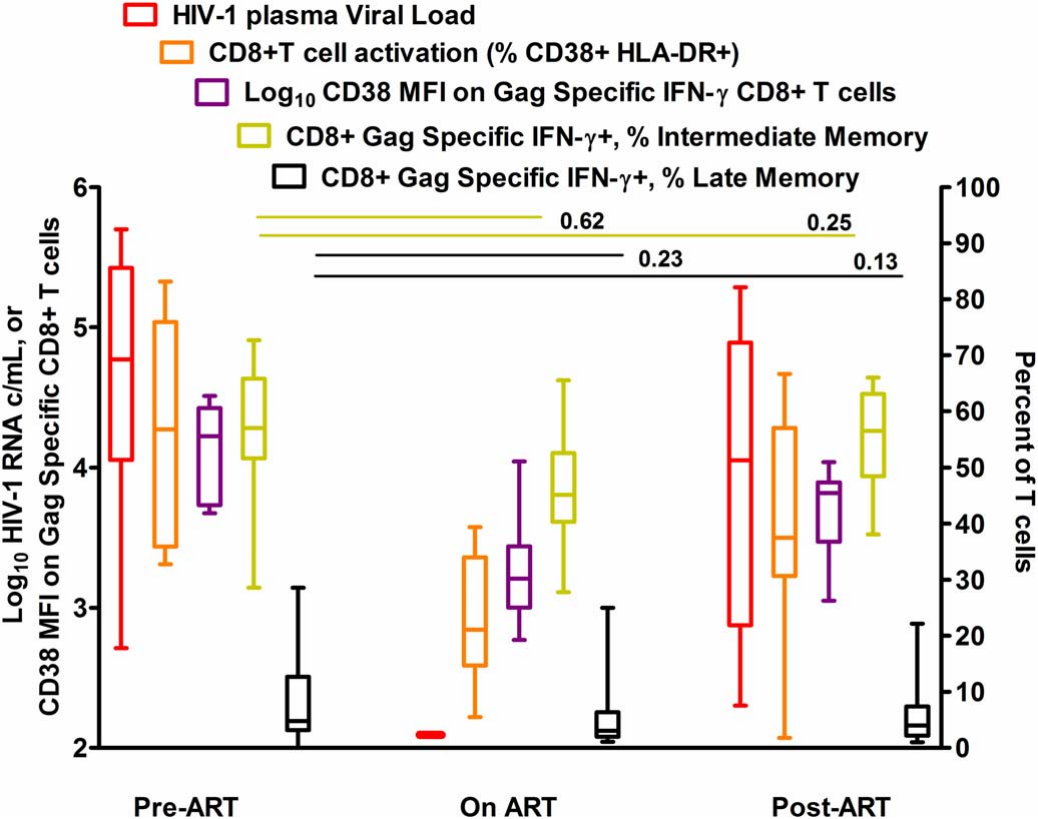
T cell activation declines during anti-retroviral therapy but maturation profile of Gag specific CD8+ T cells does not change HIV-1 RNA levels, total CD8+ T cell activation levels, HIV-1 specific CD8+ T cell activation levels and the proportion of HIV-1 Gag specific IM and LM CD8+ T cells at visit 1 (Pre-ART), visit 2 (On ART) and visit 3 (Post-ART). P-values for changes in viral load, Total and HIV-1 specific CD8+ T cell activation are shown in Table 1. HIV-1 RNA, total CD8+ T cell activation levels and HIV-1 gag specific CD8+ T cell activation levels all declined after initiation of ART. Black bars display p-values for a test of change in HIV-1 Gag specific CD8+ T cell IM and LM fractions from visit 1 to visit 2, and visit 3. Neither the Gag specific IFN-γ+ CD8+ IM or LM pool changed significantly from pre-therapy levels after therapy was initiated (On ART), or later halted (Post-ART).

Although there was a sharp decline in the level of activation (CD38 MFI) on HIV-1 Gag specific CD8+ T cells, there was no significant drop in the fractional size of the CD8+ T cell HIV-1 Gag specific IM CD27+CD28− population (Fig. 7), or increase in the fractional size of HIV-1 Gag specific LM CD8+ T cell pool. Once therapy was halted, CD38 expression increased on Gag-specific CD8+ T cells, while there was no net change in the fractional size of IM or LM sub-populations from pre-treatment (Fig. 7).

## Discussion

In an effort to understand the relationship of two key features of the early T cell response to HIV-1 we examined the simultaneous expression of both activation and differentiation markers on total and antigen-specific T cells from treatment naïve adults in early HIV-1 infection before, during and after anti-retroviral therapy. Our data suggests that there may be a link between the activation and maturation stage of CD8+ T cells. The skewed, less differentiated maturation profile of HIV-1-specific T cells was more pronounced at higher CD8+ T cell activation levels. We also found that increased numbers of fully differentiated HIV-1-specific CD8+ T cells associated with higher CD4+ T cell counts suggesting that the presence of mature Gag-specific CD8+ T cells may be protective. However the HIV-1 Gag-specific T cell differentiation profile was not readily altered by suppression of T cell activation by anti-retroviral treatment.

We compared CMV- and HIV-1-specific responses in early infection. Previous studies of differences between CMV- and HIV-specific-responses have suggested that the failure of HIV-specific cells to reach a more differentiated phenotype may explain their inability to fully control HIV replication [12], [17]. The magnitude of CD8+ IFN-γ+ and IFN-γ+IL-2+ T cell responses to CMV pp65 were considerably higher than those for HIV-1 Gag. CMV pp65-specific CD8+ T cells had a greater proportion of the more mature LM (CD27−CD28−) cells compared with Gag-specific CD8+ T cells. The level of activation on all subsets of pp65-specific cells was much lower than on Gag-specific cells. These data suggest that a low activation state, a high magnitude of CMV specific IFN-γ and IL-2 responses, and a high proportion of mature CMV specific CD8+ T effector cells may be important features in the control of CMV.

It has been suggested that distinct T cell maturation profiles, associated with responses to different viruses, are established for the purpose of generating memory or maintaining latency [9]. A recent report suggested that protective Class I B*27 HIV-1 KK10-specific CD8+ T cells have a intermediate differentiation phenotype (CD27+CD28−CD45RA-CCR7-) that was indistinguishable from CD8+ T cells specific for other less-protective HIV-1 epitopes [18]. This may indicate that an intermediate differentiation phenotype can be associated with protection. That said, in agreement with our finding that a more differentiated HIV-1 specific CD8+ T cell response is associated with higher CD4 counts two recent clinical studies have suggested that more differentiated CD8+ T cell responses are associated with lower HIV-1 viral load set point, or long-term non-progressor status in HIV-1 disease [10], [11]. Lower CD8+ T cell activation may mark or induce a state of less differentiated total and CD4+ T cell responses. This in turn may amount to a lower fraction of IM CD4+ T cells expressing CCR5 and lower cell turn-over, rendering the CD4+ T cell population less susceptible to infection. The reverse state - of higher CD8+ T cell activation - may associate with increased CD4+ T cell loss due to cytokine forced expansion of EM CD4+ T cells into an IM pool. This stimulation, in the absence of cognate antigen could lead to apoptosis, or direct viral infection and cell death.

We found that patients with the highest numbers of EM Gag-Specific CD4+ T cells had the lowest levels of T cell activation, suggesting that – in contrast to our findings on CD8+ T cell responses – that a less differentiated CD4+ T cell response may be associated with better clinical status. Our data is consistent with a report suggesting a less differentiated CD4+ T cell response marks long-term non-progression in HIV disease, a state of low CD8+ T cell activation [19].

Repeated stimulation of Gag-specific T cells, by persistent HIV-1 replication, may induce high levels of activation and block maturation into a mature effector phenotype. Based on our model linking activation to maturation impairment, we reasoned that in the absence of repeated antigen stimulation the proportion of Gag-specific T cells with a low activation, LM phenotype may increase. We therefore examined maturation and activation following anti-retroviral therapy and reduction of circulating virus to undetectable levels. While a significant decline in activation levels on all CD8+ T cell subsets was apparent, reduced antigen load had different effects on total, CMV- and HIV-1-specific T cell differentiation profiles. Following therapy, the CMV-specific CD4+ IM T cell pool did expand as the LM pool shrunk. This suggests that HIV-1 viremia, or generalized immune activation may influence the differentiation profile of CMV specific CD4+ T cell responses. Among total CD8+ T cells there was a significant reduction in the IM and a corresponding increase in the LM fraction. As activation declines the total IM CD8+ T cell fraction may mature, or die by apoptosis, shifting the population to an LM profile. In contrast, despite a significant reduction of activation levels on Gag-specific CD8+ T cells, there was no corresponding shift in the maturation profile of Gag specific CD8+ T cells. Taken together, these data suggest that high activation and viral load does not prevent the maturation of Gag-specific cells, although these effects may be apparent on T cells of other specificities. The Gag-specific T cells that we measured may have already become replicatively senescent due to repeated stimulation [16].

The mechanism by which maturation and activation are associated prior to treatment is not clear from our results. The relative paucity of LM HIV-specific CD8+ T cells may be due to increased susceptibility of these to apoptosis, even after ART suppression of activation. That said, the failure of CD8+ T cells to mature may result from manipulation of signaling pathways in responding HIV-1 specific CD8+ T cells by a viral product, such as secreted Nef, which has been shown to upregulate PD-1 [20]. Indeed PD-1, which is associated with anergy of antigen-specific cells, is upregulated on HIV- but not CMV-specific CD8+ T cells [21] and could prevent maturation to an effector cell type. It has also been suggested that lack of IL-2 production by HIV-1-specific CD8+ T cells may explain their inability to down-regulate CD27 [17]. Recently it was shown that CD27 down-regulation can be blocked by lack of expression of its ligand, CD70 [22] which requires both antigen and cytokines, including IL-2 for expression. In our study the proportion of Gag-specific CD8+ IL-2 expressing cells was very low (Fig. 3) supporting the idea that the absence of IL-2 producing HIV-1-specific T cells may be unable to prevent CD27 down-regulation [22].

We observed that HIV-specific T cells are predominantly immature and highly activated at early stages of infection. Although activation is reduced by anti-retroviral therapy immature Gag-specific cells continue to predominate. In a previous report we demonstrated that initiation of anti-retroviral therapy within the first month of HIV-1 infection associated with improved viral control and clinical outcomes once therapy was halted, compared to those who start anti-retroviral therapy one month or later into infection [23]. In our current study, persons initiated therapy no earlier than 2 months after acquiring HIV-1, by which time a sub-optimal HIV-1 specific T cell differentiation profile was established. It would be of interest to examine the activation and maturation profile of cells from individuals treated within one month of infection. Earlier intervention with anti-retroviral therapy may lower T cell activation during very early infection, alter the T cell differentiation phenotype of responding CD8+ T cells, or both, in a manner which confers enhanced T cell mediated control of HIV-1.

## Materials and Methods

### Overview

Thirteen treatment naïve adults in early HIV infection were studied for T cell responses and phenotypes at 1) study entry (within 2–4 months of acquiring infection), 2) after having reached complete virologic suppression on a first anti-retroviral regimen (defined as 2 nucleoside reverse transcriptase inhibitors, and at least 1 protease inhibitor and/or 1 non-nucleoside reverse transcriptase inhibitor) and 3) several months after having halted anti-retroviral therapy.

#### San Francisco Recent HIV-1 Infection Cohort

All specimens were drawn from persons enrolled in the Options study of early HIV-1 infection conducted in a university based research clinic. Among those enrolled in OPTIONS, approximately 90% are within 6 months of acquiring HIV-1 infection [24]. Patients were determined to be in early HIV-1 infection via an algorithm employed by the Acute Infection and Early Disease Research Program. This algorithm employs information on serial HIV-1 antibody testing, de-tuned EIA scores [25], [26], RNA PCR detection, HIV-1 protein western blot banding patterns and self reported risk behaviors to estimate time since infection. Only persons estimated to be within 1 year of infection are enrolled in the parent cohort (OPTIONS), and most patients are within 3 to 6 months post-infection at the time they enroll. Information on estimated length of infection for study participants may be found in Table 1. All participants gave written, informed consent using protocols approved by the Committee on Human Research, University of California, San Francisco.

#### Cell Specimens

PBMC were isolated, cyropreserved and stored by the UCSF/ARI AIDS Specimen Bank, then transported to the Core Immunology Laboratory for analysis. Cryopreserved PBMCS were rapidly thawed in to warm RPMI 1640 with 10% fetal bovine serum (UCSF Cell Culture Facility) and counted using the Viacount assay on a Guava Personal Cell Analysis system (Guava Technologies). For Cytokine Flow Cytometry (CFC) assays PBMC were re-suspended at 1×10^6^–2×10^6^ cells/mL in the same medium, and rested overnight in slanted 15-mL conical tubes with loosened caps, in a CO_2_ incubator at 37°C. For phenotyping assays cells were washed in FACS buffer (PBS with 1% Bovine Serum Albumin, Sigma Aldrich) for staining the same day.

#### T cell activation and maturation

Thawed PBMCS were surfaced stained with the following combination of fluorescently labeled antibodies: CD3 Pacific Blue, CD4-AmCyan, CD8-Alexa 700, CD38-PE-CY7, CD27-APC, CD28-APC-CY7, and HLADR FITC (all BD Biosciences) in the presence of 5 µg/ml ethidium monoazide bromide (EMA, Invitrogen). After a 50 minute incubation in the dark at 4°C cells were exposed to a 40-W fluorescent light for 10 min at room temperature to cross-link the EMA. FMO (Fluorescent minus-one) controls were run with each experiment to ensure that breakdown of tandem conjugates (e.g. APC-CY7 to APC) was not occurring and to assist with setting gates. Following staining, PBMCs were washed in FACS buffer, fixed in 0.5% formaldehyde (Polyscience), stored at 4°C until analysis.

#### Antigen Specific CD4+ and CD8+T cells

Thawed and rested PBMC were stimulated with overlapping peptide pools (all 15mers overlapping by 11 aa) in the presence of 10 µg/ml Brefeldin A (Sigma Aldrich) for 18 hours at 37°C. Peptide pools were HIV-1 SF2 GAG (4.8 µg/mL, SynPep) and CMV pp65 (4.25 µg/mL, SynPep). Unstimulated cells were run in parallel as a negative control for each subject. Following stimulation PBMCs were treated with 2 mmol/L EDTA, washed in PBS, then stained with anti-CD4, CD8, CD38, CD27, and CD28 in the presence of EMA, as described above. Cells were then washed in FACS buffer, fixed and permeabilized by a 10 minute incubation in FACS lyse, and a 10 minute incubation in FACS Perm (both from BD Biosciences) before staining with fluorescently conjugated antibodies: IFN-γ-FITC, IL-2-PE and CD3-Pacific Blue (all BD Bioscience). Cells were washed, re-suspended in FACS buffer, stored at 4°C until analysis.

#### Flow Cytometry

All samples were run on a customized BD LSR II Flow cytometer within 18 hours of staining. Rainbow beads (Spherotec) were used to standardize instrument settings between runs. Between 900,000 and 1 million lymphocytes were collected for each sample. Data was compensated and analyzed by using Flowjo Software (Treestar Inc). The gating strategies used to define CD4+ and CD8+ T cell maturation and activation populations, to define antigen-specific T cells, and to subset these cells for activation and maturation markers are shown in figure 1. We defined a T cell response as the percent of cells expressing cytokine in response to HIV Gag or CMV pp65 after subtraction of background signal. The proportion of CD8+ T cells responding to antigen stimulation varied from 0 to 7.5% (median IFNγ+ response 0.46% to Gag and 1.6% to pp65) and in some cases insufficient cytokine positive events were obtained to determine the level of activation or maturation markers. CD4+ T cells responses were low for some subjects (median IFNγ+ response 0.072% to Gag and 0.15% to pp65). We restricted the analysis of maturation markers to responses where greater than 100 IFNγ+ events were collected. As a result we do not present subset analyses of the IFN-g\IL-2 responding populations, nor do we present the expression of CD38 MFI on antigen-specific CD4+ T cell responses as the IM and LM populations were scarce. The activation state of antigen specific cells was measured by examining the median fluorescence intensity (MFI) of CD38 on each differentiation stage. In this analysis we measured CD38 MFI expression on the IFN-γ expressing CD4+ or CD8+ T cell population. We assigned the CD8+ T cell differentiation stages according to the scheme proposed by Appay as Early Memory (EM) for CD27+CD28+, Intermediate Memory (IM) for CD27+CD28- and Late Memory (LM) for CD27−CD28− CD8+ T cells [12], [27]. For CD4+ T cells the categories were the same, except that the Intermediate Memory (IM) CD4+ T cells are defined as CD27−CD28+.

#### Statistical Analysis

All statistical analyses were performed in the SAS System Version 9.1 for Windows XP. Non-parametric statistical tests were employed in all cases. Spearman rank correlations were generated for all correlation tests. Signed Rank tests were used to test significance of change in values between study time-points. The Wilcoxon Two Sample test was used to compare different values at a given study time-points.
